# Distributed learning for heterogeneous clinical data with application to integrating COVID-19 data across 230 sites

**DOI:** 10.1038/s41746-022-00615-8

**Published:** 2022-06-14

**Authors:** Jiayi Tong, Chongliang Luo, Md Nazmul Islam, Natalie E. Sheils, John Buresh, Mackenzie Edmondson, Peter A. Merkel, Ebbing Lautenbach, Rui Duan, Yong Chen

**Affiliations:** 1 Perelman School of Medicine, The University of Pennsylvania, Philadelphia, PA USA; 2 Division of Public Health Sciences, Department of Surgery, Washington University in St. Louis, St. Louis, MO USA; 3 Optum Labs, UnitedHealth Group, Minnetonka, MN USA; 4 Harvard T. H. Chan School of Public Health, Harvard University, Boston, MA USA

**Keywords:** Risk factors, Data integration

## Abstract

Integrating real-world data (RWD) from several clinical sites offers great opportunities to improve estimation with a more general population compared to analyses based on a single clinical site. However, sharing patient-level data across sites is practically challenging due to concerns about maintaining patient privacy. We develop a distributed algorithm to integrate heterogeneous RWD from multiple clinical sites without sharing patient-level data. The proposed distributed conditional logistic regression (dCLR) algorithm can effectively account for between-site heterogeneity and requires only one round of communication. Our simulation study and data application with the data of 14,215 COVID-19 patients from 230 clinical sites in the UnitedHealth Group Clinical Research Database demonstrate that the proposed distributed algorithm provides an estimator that is robust to heterogeneity in event rates when efficiently integrating data from multiple clinical sites. Our algorithm is therefore a practical alternative to both meta-analysis and existing distributed algorithms for modeling heterogeneous multi-site binary outcomes.

## Introduction

Starting from the 2010s, the adoption of Electronic Health Record (EHR) systems grows rapidly in the United States. A large range of detailed clinical data, including medications, laboratory test results, disease status, and treatment outcomes, are available to facilitate research. The real-world data (RWD), including EHRs, claims, and billing data among others, have become an invaluable data source for comparative effectiveness research (CER) during the past few years^1,2^. Synthesis of the RWD stored electronically in the EHR systems from multiple clinical sites provides a larger sample size of the population compared to a single site study^3^. Analyses using larger populations can benefit the accuracy in estimation and prediction. The integration of research networks inside healthcare systems also allows rapid translation and dissemination of research findings into evidence-based healthcare decision making to improve health outcomes, consistent with the idea of a learning health system^4–9^.

In the past few years, several successful networks have been founded and become beneficial to multicenter research. One of them is the Observational Health Data Sciences and Informatics (OHDSI) consortium^10^. OHDSI was founded for the primary purpose of developing open-source tools that could be shared across multiple sites. OHDSI developed the Observational Medical Outcomes Partnership (OMOP) Common Data Model (CDM) for data standardization^11^. The OMOP allows each institution to transform the local EHR data to the CDM’s standards. This procedure makes it feasible for the researchers to develop methods that can be simultaneously applied to the datasets from many institutions. The conversion and standardization of the data format decrease the probability of translation error and also increase the efficiency of data analysis. Another successful network is the National Pediatric Learning Health System (PEDSnet), a National Pediatric Learning Health System, within the PCORnet system^12,13^. This network contains eight large pediatric health systems in the US. Comprising clinical information from millions of children, PEDSnet offers the capacity to conduct multicenter pediatric research with broad real-world evidence. Other significant efforts include Sentinel System, which is a multi-site network of a national electronic system for monitoring performance of FDA-regulated medical products^14^ and the Consortium for Clinical Characterization of COVID-19 by EHR (4CE)^15^, which is an international consortium for electronic health record (EHR) data-driven studies of the COVID-19 pandemic, among others.

In multi-center studies, maintaining privacy of patient data is a major challenge^16–19^. Due to data privacy policies, directly sharing patient-level data, especially demographic, comorbidity, and outcome data, is restricted and poorly feasible in practice. The Health Insurance Portability and Accountability Act of 1996 (HIPAA) introduced a privacy rule to regulate use of protected health information (PHI) often found in EHRs, requiring de-identification of PHI before use in biomedical research^17^. De-identified PHI has been shown to be susceptible to re-identification, causing concern among patients^20,21^.

In light of patient privacy concerns, many multicenter studies currently conduct analyses by combining shareable summary statistics through meta-analysis^22–24^. While relatively simple to use, meta-analysis has been shown to result in biased or imprecise estimation in the context of rare outcomes, as well as with smaller sample sizes^25^. Other than meta-analysis, several distributed algorithms have been developed and considered in studies with multi-site data. In these distributed algorithms, a model estimation process is decomposed into smaller computational tasks that are distributed to each site. After parallel computation, intermediate results are transferred back to the coordinating center for final synthesis. Under this framework, there is no need to share patient-level data across sites. For example, GLORE (Grid Binary LOgistic Regression) was developed for conducting distributed logistic regressions^26^, and WebDISCO (a Web service for distributed Cox model learning) was developed to fit the Cox proportional hazard model distributively and iteratively^27^. Both algorithms have been successfully deployed to the pSCANNER consortium^28^. Through iterative communication of aggregated information across the sites, these two algorithms provide accurate and lossless results, which are equivalent to fitting a model on the pooled data from all sites. However, in practice these methods can be time-consuming and communication-intensive due to the need for iteratively transferring data. To overcome this limitation, non-iterative privacy-preserving distributed algorithms (specifically, one-shot algorithms, which only require one round of communications across sites) for logistic regression (termed as ODAL) and Cox model (termed as ODAC) through the construction of a surrogate likelihood have been proposed^25,29,30^.

However, all of the aforementioned distributed algorithms rely on the assumption that data across clinical sites are homogeneous. This assumption is often not reflecting the reality in biomedical studies because often there are intrinsic differences across clinical sites in terms of population characteristics, types of interventions, data collection procedures, and so on. Ignoring heterogeneity across clinical sites can induce biases in estimating associations between the exposures of interest and outcomes^16,31^. There was a limited amount of effort in addressing this issue. For binary outcomes, to account for the situation that some studies are substantially different from the others in multi-site studies, a Robust-ODAL algorithm, built on robust statistics in data aggregation, was proposed^32^. Duan et al. (2021)^33^ proposed a framework of distributed inference for heterogeneity-aware distributed algorithms. By parametric modeling of the data generating mechanism of all data sites, a density ratio tilting technique was developed in characterizing the impacts of between-site heterogeneity and an efficient score function was developed to reduce the impacts of the between-site heterogeneity.

Despite the existing limited efforts on accounting for heterogeneity, all of them are based on *fully* parametric models, which require full characterization of data generating mechanism of the data. In this paper, we plan to develop an alternative distributed algorithm based on models that allow site-specific effects, without the need for specification of the distribution of the site-specific effects, which brings robustness to statistical inference. We devise our algorithm to be communication-efficient, which only requires one-round of communication from the collaborative sites.

**Fig. 1:**
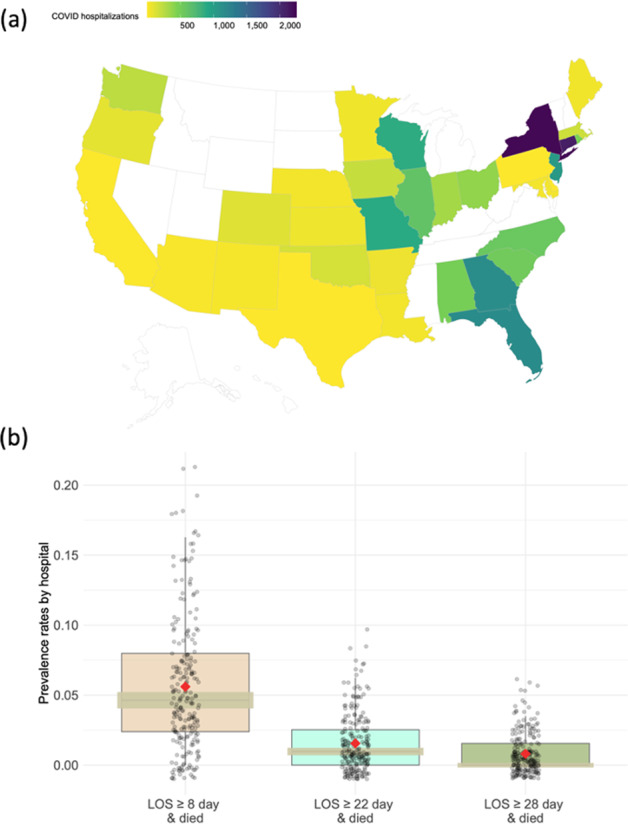
Summary of real-world data from 230 sites. **a** COVID-19 cases distribution: number of COVID-19 hospitalizations included in the study are represented across 47 states created by open-source R package *usmap*^34^ (https://cran.r-project.org/web/packages/usmap/usmap.pdf) (**b**) Box plots of the prevalence rates of composite outcomes of 230 hospitals.

Our motivating example is a claims data derived from the insurance claims of 14,215 patients who were diagnosed with COVID-19 prior to June 29, 2020. Containing demographic, diagnosis, and procedural codes, the claims data are collected from 230 sites documented in the UnitedHealth Group Clinical Research Database. There is a substantial difference in clinical practices across these sites due to such factors as geographical variability in disease patterns, variations in patients’ characteristics, and regional differences in practice patterns. Specifically, large variation in the COVID-19 hospitalization distribution exists across 47 states in the U.S. (Fig. 1(a), created by open-source R package *usmap*^34^). The rate of the interested outcome, defined by combining both hospitalizations (days) and the status of patients being expired, ranges from <1% to 6% across the 230 sites as shown in Fig. 1(b). Therefore, developing methods to account for the heterogeneity in the data is especially needed when analyzing multi-site data within the networks.

To fill the above methodology gap, in this paper, we develop a privacy-preserving distributed pairwise conditional logistic regression (dCLR) algorithm. The proposed dCLR algorithm accounts for between-site heterogeneity by a construction of pairwise likelihood, and facilitates data integration by efficient communication (i.e., only requires one round of communication of aggregated information from collaborative sites). Instead of using the standard conditional logistic regression model by Breslow and Day^35^, the pairwise conditional likelihood^31^ has the advantage of computational advantages by reducing the computational cost from a permutation (i.e., n! where n is the sample size in a site) to all possible pairs (i.e., O(n^2^)). Based on the seminal work on surrogate likelihood proposed by Jordan et al. (2019)^36,37^, we construct a surrogate pairwise likelihood through approximating the target pairwise likelihood by its surrogate. The construction of pairwise likelihood avoids estimating the site-specific intercepts in the regression model, and leads to robust estimation of regression coefficients. Different from existing work on surrogate likelihood^36,37^ where data are assumed to be homogeneous, to deal with heterogeneous data, we use U-statistics theory to show that the proposed surrogate pairwise likelihood leads to a consistent and asymptotically normal estimator, and is asymptotically equivalent to the maximum pairwise likelihood estimator based on the pooled data. We evaluate the empirical performance of the proposed method through simulation studies and a study of the risk factors associated with increased length of stay outcomes from 14,215 patients across 230 clinical sites admitted with COVID-19.

## Results

### The dCLR algorithm is privacy-preserving, heterogeneity-aware, and communication-efficient

**Fig. 2:**
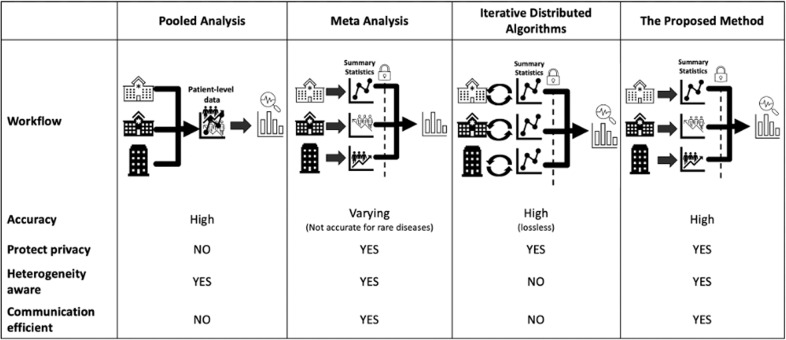
Existing methods comparison. Comparisons between pooled analysis, meta-analysis, iterative distributed algorithms, and the proposed method. The proposed method can retain high accuracy when estimating association between exposures and outcome of interest. In addition, the proposed method can handle heterogeneity across the sites and protect patient privacy with efficient communication.

Figure 2 shows the comparisons between the pooled analysis, meta-analysis method, iterative distributed algorithms, and the proposed method from various aspects. Accuracy is evaluated through mean squared error (MSE) and bias to the true value: the smaller the MSE or bias is, the better the accuracy is. Privacy is evaluated based on if the method is an aggregated data-based approach without sharing patient-level information. Heterogeneity refers to the different disease prevalence values or baseline risks, which can be evaluated by calculating the variance or the range of the intercepts of the model^32,38^ The evaluation of communication is through the number of rounds of transferring aggregated data across sites and the number of digits to be communicated within each round. The proposed method, which is based on the surrogate pairwise likelihood approach, can retain high accuracy in estimated model parameters, protect patient privacy, handle heterogeneity, while being communication efficient.

### The dCLR algorithm provides highly accurate estimation of model parameters

In our simulation study, we consider a setting where a binary outcome is associated with two risk factors. Three total numbers of sites were simulated (i.e., five, twenty, and two hundred clinical sites in total). We simulated three scenarios of the disease prevalence to mimic the real-world common disease and relative rare disease. For simplicity, we only present the results for the estimation of one of the two risk factors’ coefficients and the results for the other coefficients are similar.

**Fig. 3:**
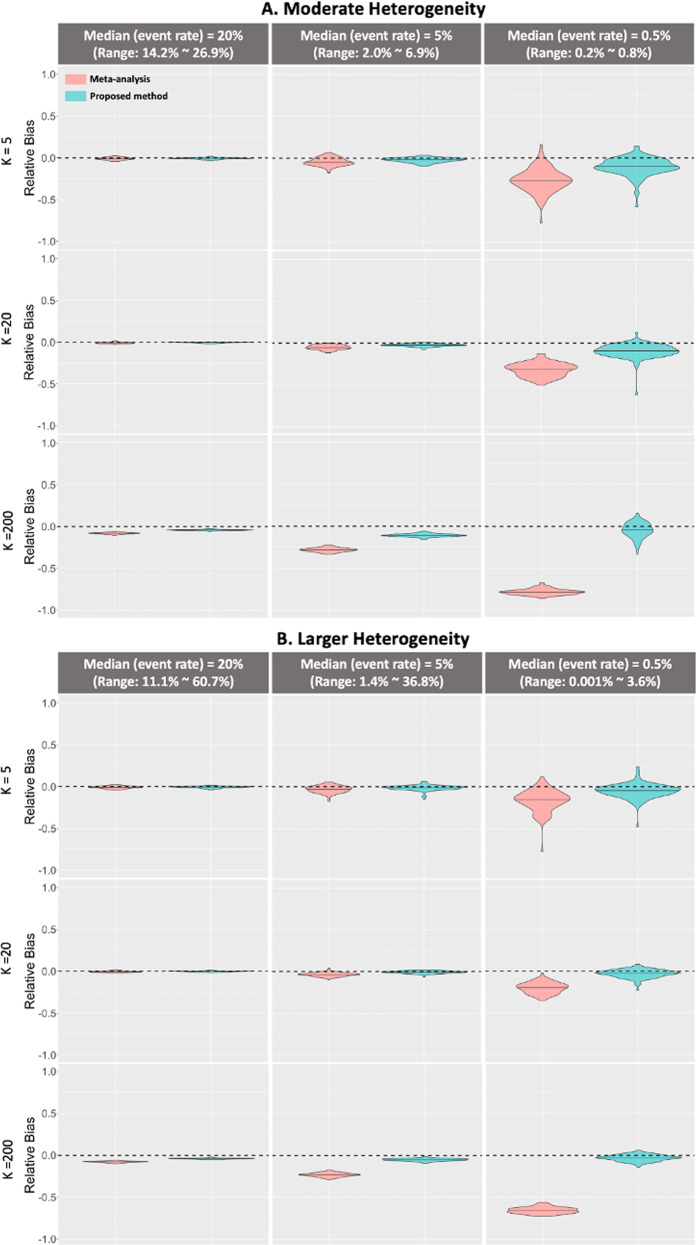
Simulation study results. Comparison between the relative bias of meta-analysis method (pink) and the proposed dCLR algorithm (cyan). Upper panel: relative bias of the continuous risk factor’s coefficient estimation compared with the pairwise likelihood method (gold standard) under three scenarios (i.e., median prevalence 20%, 5%, and 0.5%) with moderate heterogeneity in prevalence when the total number of sites is 5, 20, or 200. Lower panel: relative bias of the continuous risk factor’s coefficient estimation compared with the pairwise likelihood method (gold standard) under three scenarios with median prevalence 20%, 5%, and 0.5% with larger heterogeneity (i.e., larger prevalence range than upper panel) when the total number of sites is 5, 20, or 200.

Figure 3 shows the violin plot of the relative bias compared with the pairwise likelihood method under different numbers of sites and event rates. Upper panel represents the moderate heterogeneity in the prevalence rates and the lower panel represents a larger heterogeneity. The first row in each panel is for the results when the total sites number is five, the second row is for the setting when total sites number is twenty, and the third row is for the setting when total sites number is two hundred. The black dashed line represents zero relative bias compared with the gold standard method. From the figure we observe that for all scenarios, the proposed method obtains almost the same or smaller relative bias compared with meta-analysis. Importantly, as the event rate decreases under both less and more heterogeneous cases, the meta-analysis estimator is observed to have larger bias. When the event rate is <5%, the relative bias of the proposed estimator is 30% smaller than that of the meta-analysis estimator. In summary, the proposed method can provide better performance than the meta-analysis estimators to handle the heterogeneity across the clinical sites when the event is rare.

### The dCLR algorithm can integrate data across *heterogenous* clinical sites

Administrative claims data for 14,215 hospitalized patients who were diagnosed with COVID-19 prior to June 29, 2020, from 230 clinical sites were used to estimate the association between clinical-and-demographic covariates (i.e., age, sex, line of business, and Charlson comorbidity index) and therapeutic patient outcomes.

**Fig. 4:**
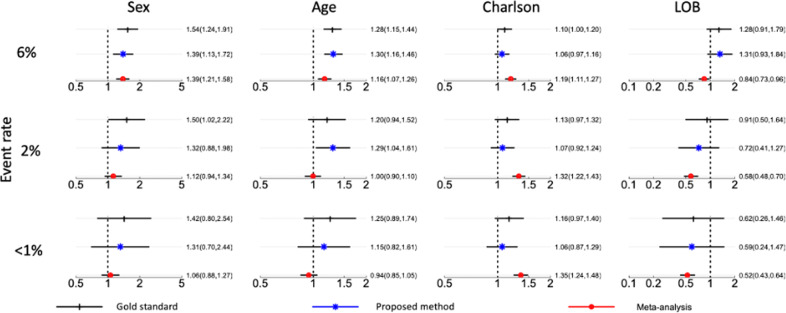
Real-world data analysis results. Point estimates and 95% confidence intervals (CI) for the association (in odds ratio scale) between the LOS (i.e., length of stay) and covariates (i.e., sex, age, Charlson score, line of business, from left to right). Each row represents an event rate of the outcome: 6%, 2%, and <1% from top to bottom. Each column represents the estimation of the covariate.

We primarily focus on estimating and comparing parameter estimates by the proposed method and the meta-analysis method. We stress that the parameter estimates need to be interpreted with caution since the effects’ magnitudes or directions might be misleading without adjusting for potential confounders in the model. Figure 4 illustrates the results obtained by the pairwise likelihood method (i.e., gold standard method with pooled patients’ patient-level data), the proposed method, and meta-analysis. As the prevalence rate decreases (i.e., in rare events), the proposed method outperforms meta-analysis in terms of estimating parameters. Specifically, the odds ratio (OR) of the proposed method remains closer to that of the gold standard approach, compared with the OR of meta-analysis. The proposed estimates have a relative bias <9% when the event rate is <1%, whereas the meta-analysis estimates have a relative bias at least 10% higher than that of the proposed method. With the bootstrap method with 100 replications, the differences with respect to the gold standard method (black, top) between the proposed algorithm (middle, blue) and the meta-analysis method (bottom, red) are all statistically significant with the p-values smaller than 0.001 across all settings for all covariates. This observation matches with that of the simulation study.

Besides, meta-analysis underestimates variance (or standard error of estimates) leading to far narrower confidence intervals relative to those of the gold standard method, especially for rare events. Ignoring between-and-within sites correlation in meta-analysis is likely to induce bias and underestimate uncertainty in parameter estimates leading to conflicting inference about the testing of significance of the effect size. For example, 95% confidence intervals of ORs for Charlson score based on meta-analysis does not contain OR value of one implying its significance, which is inconsistent with the inference based on the gold standard method. In contrast, the proposed method produces comparable inferences to the gold standard method.

## Discussion

In this paper, we proposed a robust privacy-preserving distributed algorithm for modeling binary outcomes while accounting for heterogeneity across clinical sites. The proposed method only requires one round of communication of aggregated data. Our algorithm provides an estimator that is robust to heterogeneity in event rates. In simulations, the proposed method is shown to have higher accuracy than meta-analysis when the outcome is relatively rare, suggesting its utility in a rare-event context.

There are several advantages of our proposed algorithm compared to existing methods for privacy-preserving data analysis. Relative to meta-analysis, our method accesses patient data at a higher granularity while requiring minimal additional effort to institute. For multi-site studies operating under a common data model, such as OHDSI, analyses using our method can be carried out at individual sites concurrently without the need for any site-specific modifications. In addition, there are several benefits of using our method compared to existing distributed algorithms. First, the proposed algorithm does not require iterative communication across the sites, leading to the reduction in communication costs and administrative efforts. Secondly, to implement the proposed method, the access of patient-level data is required only at a single site. For the other sites within the network, the aggregated information will be used instead of patient-level data transfer across the sites to construct the surrogate pairwise likelihood function. Given the understandable privacy- and proprietary-related sensitivities health systems have to provide “outside” collaborators with access to patient-level data, limiting the need to use such data to only one site would be extremely beneficial to a multi-site project in terms of feasibility, costs, and time. Thirdly, by eliminating the site-specific parameter in the conditional logistic regression model, the proposed method can handle the heterogeneity across the sites *without modeling their distribution*. In this paper, we specifically focused on the binary outcome to illustrate the proposed method based on the motivating example. Generally, the proposed algorithm can consider the models within the generalized linear model (GLM) and further to the semi-parametric extension of GLM, for example, the semiparametric proportional likelihood ratio proposed by Luo and Tsai (2012)^38^. With the conditioning technique, the proposed distributed algorithm does not require parametric assumption on the baseline distribution of patient characteristics. The aforementioned advantages of our algorithm comes with the price of higher computational cost, as the algorithm involves computation of likelihood constructed by all pairs of patients within a site. To alleviate this limitation, we implemented an algorithm with R calling C, which is about 50 times faster than using the R programming language alone. We have demonstrated its applicability to real-world data with a large number of patients in a database with 14,215 patients across 230 clinical sites.

Nevertheless, the proposed method has several limitations that require further investigation and evaluation. First, the proposed pairwise likelihood function can only handle the heterogeneity of the intercepts (i.e., site-specific effects) in the regression model. There exist other types of data heterogeneity, such as heterogeneous effects of the predictors, and heterogeneity in data structure. To handle these types of heterogeneity, further development of distributed algorithms is needed. Secondly, in the proposed dCLR algorithm, we focused on the heterogeneous baseline risks (i.e., prevalence values) but did not investigate the intra-site heterogeneity where subpopulations within a site may have different outcomes. To account for the intra-site heterogeneity, there are several strategies. For example, we can include interaction terms between dummy variables for the subpopulations (such as ICU patients vs other patients) and the covariates in other to account for heterogeneous effect sizes of the covariates. Further, high-dimensional extensions of the pairwise likelihood, such as Ning et al. 2017^39^, with added interaction terms among covariates should be considered in accounting for intra-site heterogeneity. Thirdly, the proposed dCLR algorithm is considered privacy-preserving because there is only one round communication of aggregated data from the clinical sites needed. The aggregated data are made available only to the participants of the collaborative investigation. Nevertheless, the release mechanism of our aggregated data has not been rigorously studied to ensure the privacy-preserving criteria such as k-anonymity or differential privacy have been met. The aim of k-anonymity is to protect against the risk of re-identification, which arises from linking potential quasi-identifiers (i.e. combinations of patient’s characteristics in our study) to external sources. For the proposed dCLR algorithm, it can potentially meet the k-anonymity requirement if all the cell counts in the aggregated data are not sparse. In the future collaborative studies using dCLR, we suggest the data contributors review the aggregated data to avoid sparse cells before sending them to other sites. In the future, we plan to further extend our algorithms under the abovementioned heterogeneity settings and assess the probability of privacy leakage and improve our dCLR algorithm using approaches such as differential privacy and multiparty homomorphic encryption.

In the future, we also plan to extend our algorithm in several aspects. We plan to develop methods for other types of outcomes, such as time-to-event data and count data. Recently, a number of one-shot distributed algorithms have been developed for analyzing heterogeneous multi-site time-to-event data allowing for site-specific baseline hazard functions, count data allowing for site-specific over-dispersion parameters, and zero-inflated count data with Hurdle regression^40–42^. Another strategy to account for between-site heterogeneity in multi-site data is to use hierarchical models with site-specific random effects, such as linear mixed effects model and generalized linear mixed effects model. Lossless distributed algorithms have been recently developed where the results from the distributed algorithms are identical to the results from the analysis on the pooled data. These algorithms have been applied to investigate clinical factors that impact the length of stay during hospitalization for patients admitted with COVID-19 and evaluations of performance of hospitals during pandemic^43–45^. In addition, the development of distributed algorithms to handle the missingness in the longitudinal data is also needed in the future. Additionally, we have been working on the development of the open-source software R package to implement the proposed distributed algorithm within a multicenter network. We believe that the proposed algorithm would be a useful contribution to distribution algorithms that can account for the heterogeneity across multiple clinical sites, which will ultimately advance the next generation data sharing and multi-site collaborations.

## Methods

### The proposed distributed conditional logistic regression (dCLR) algorithm

**Fig. 5:**
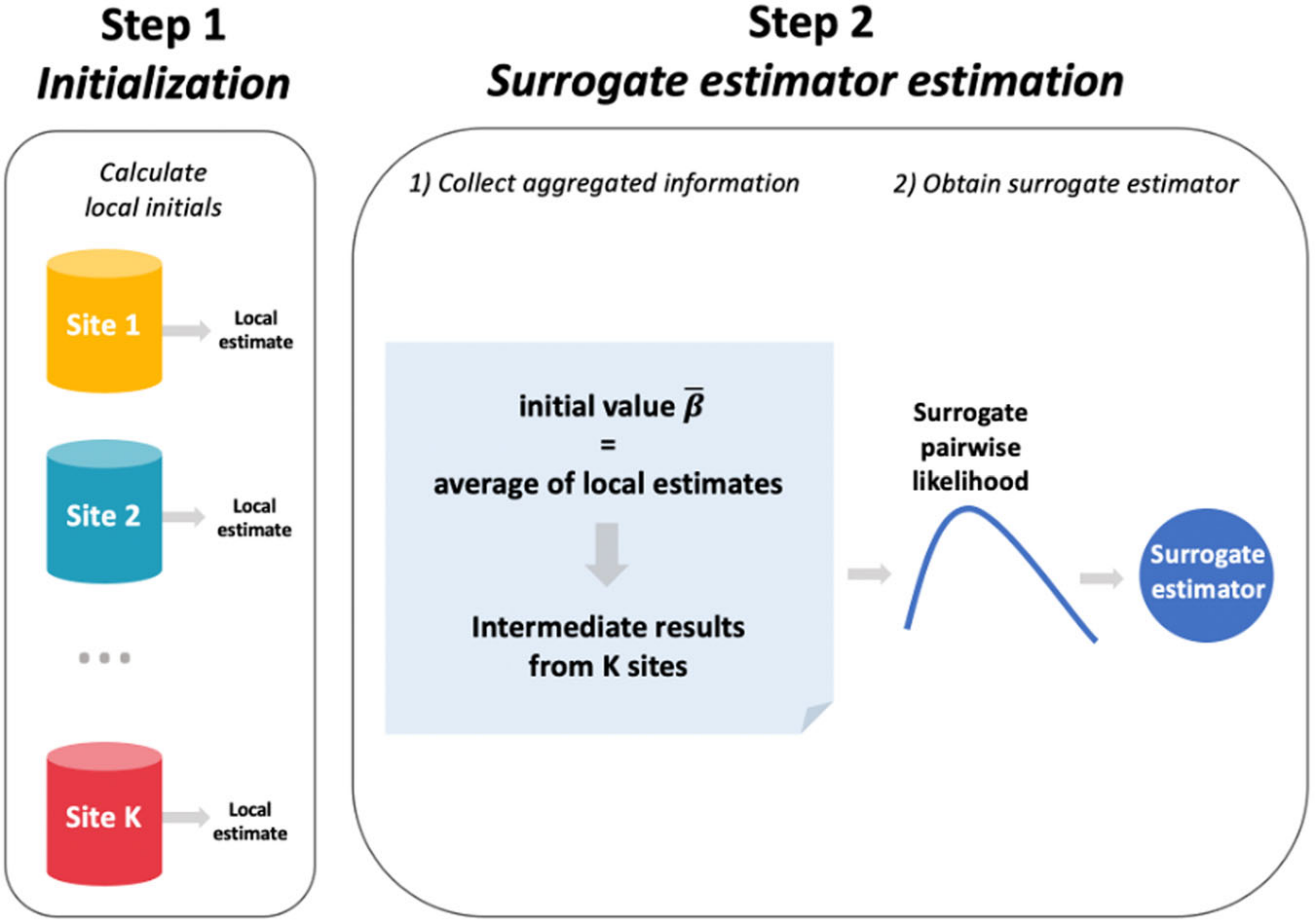
Illustration of the proposed method. Step I: Using data from each local site to estimate initial estimates and broadcast the values to calculate the weighted initial value$\bar \beta$. Step II: With the initial value $\bar \beta$, calculating the intermediate terms at each site and then transfer the results back to the local site. With the intermediate results and initial value $\bar \beta$ to construct the surrogate pairwise log-likelihood function in the local site. Maximizing the surrogate pairwise likelihood to obtain the surrogate estimator.

To handle the site-specific effect, we develop a privacy-preserving distributed pairwise conditional logistic regression (dCLR) algorithm. As shown in Fig. 5, there are two steps required to implement the proposed algorithm: initialization and surrogate estimator estimation. In the first step (i.e., initialization), each site fits a conditional logistic regression model with its own local patient-level data. Then, the sites shared the initial estimates of the parameters of interest (i.e., regression coefficients) across the collaborative sites within the network. With all the initial values, an overall initial estimate, $\bar \beta$(i.e., average or weighted average of the initial values) is calculated. In Step 2 (i.e., surrogate estimator estimation), each site first shares the intermediate results (i.e., first and second gradients of the local log likelihood function, which are calculated using the overall initial estimate $\bar \beta$). Then, the intermediate results are assembled to construct the surrogate pairwise likelihood, which is maximized by the surrogate estimator. More details on the formulas, derivation and inference of the proposed method are provided in Supplementary Method 1 and 3.

Here are the remarks of the proposed algorithm:

REMARK 1: We implemented the proposed algorithm with R calling C programming language, which is a few dozen times faster than using R programming language only. We also parallelized the running and optimization. Such implementation is necessary for the application of our algorithm to real-world settings where the number of patients in each site is relatively large.

REMARK 2: In the situation that each site is treated as the local site, each site can obtain its own surrogate pairwise likelihood estimate. These estimates can be further synthesized together with the inverse variance weighted average method to obtain an overall estimate.

REMARK 3: Given the pairwise conditioning technique used in the proposed algorithm, the proposed dCLR algorithm can handle the missingness in the data, especially some missing not at random mechanisms as outlined in our earlier investigation; see Chen et al 2015^46^; and also see Chan 2013^47^, and Ning et al 2017^39^. If the data are missing at random, imputation methods such as inverse probability weighting and imputation^48^ can be considered before implementing the dCLR algorithm.

### Numerical evaluation of the dCLR algorithm

To evaluate the empirical performance of the proposed algorithm, we conduct a simulation study to cover a wide spectrum of practical settings. We set the total number of sites, K = 5, 20, and 200. The sample size of each site is randomly sampled from a discrete uniform distribution from 800 to 1200.

We simulated three scenarios of the disease prevalence. The medians of the prevalence are 20%, 5%, and 0.5%. Specifically, the prevalence of the sites is randomly generated from a range of values as presented in Fig. 3. We also simulated two scenarios of heterogeneity under each disease prevalence to mimic moderate heterogeneous cases (upper panel) and larger heterogeneous cases (lower panel), where the prevalence ranges are larger than those of the moderate heterogeneous cases. We considered a setting where a binary outcome is associated with two risk factors, where one represents a continuous predictor (e.g., age) and the other is a binary predictor (e.g., sex, race). Under each scenario, we compared the proposed method with the pairwise likelihood method^31^, which can be treated as the gold standard and the commonly used meta-analysis. In the pairwise likelihood method, we assume that we have the access to all of the patient-level data. The simulation was conducted with 100 replications. The numerical details of the simulation settings are provided in Supplementary Method 2.

### COVID-19 data from the UnitedHealth Group (UHG) Clinical Discovery Database

In the real-world data evaluation, the data were obtained from the UnitedHealth Group (UHG) Clinical Discovery Database, which contained one or more claims from 5 million Medicare Advantage enrollees and 20 million commercially insured individuals. Suspected COVID-19 inpatient cases are manually reviewed daily by health plan clinical staff via clinical notes to determine an individual’s COVID-19 status. Each case is then manually flagged as either negative, confirmed, presumed positive, or needs clinical review.

Our analytical dataset is composed of hospitalized patients who were diagnosed with COVID-19 prior to June 29, 2020 from a single large national health insurer, which covers a broad swath of the population.

The data are from multiple EHR systems including EPIC^49^, Cerner^50^, and others. The data are recorded from 230 sites with 14,215 insured (Commercial and Medicare) patients based on the inclusion-exclusion criteria, such as age at least 18 and enrollment duration (see Figure A1 in Supplementary Figure for more details). Our objective is to develop an association model between clinical-and-demographic covariates (i.e., age, sex, line of business, and Charlson comorbidity index, which is the sum of the individual’s comorbidities weights developed by Charlson et al.^51^) and therapeutic patient outcomes. More details about the data quality are provided in Supplementary Note 4.

Outcomes are defined by combining both hospitalizations (days) and the status of patients being expired (i.e., a binary value taking value 1 if a patient is deceased or 0 otherwise). We considered three composite binary outcomes on the same cohort—14,214 patients from 230 hospitals. The binary outcomes take values 1 if the event occurs, and 0, otherwise. Here the events are defined as (a) LOS > 1 week and patient died, (b) LOS > 3 weeks and patient died, and (c) LOS > 4 weeks and patient died, respectively. Figure 1(a) shows the number of COVID-19 hospitalizations included in the study across 47 states in the U.S. and Fig. 1(b) illustrates the prevalence rates of composite outcomes by 230 hospitals. These two figures exhibit substantial variation in prevalence rates across sites. Moreover, patients admitted within the same hospital are subject to somewhat similar care, administrative facilities, and treatments provided by the same physicians. For details of the covariates, we refer to Table 1, which is the summary of characteristics of the 14,214 patients.

**Table 1:** Summary characteristics of the 14,215 patients from 230 hospitals in our population.

Number of patients	14,215
Number of hospitals	230
Patient level characteristics	
Mean age in years (Median, SD)	71.1 (73, 14.3)
Sex	
Male (%)	6,925 (48.7%)
Female (%)	7,290 (51.3%)
Mean Charlson score (Median, SD)	3.4 (3.0, 3.0)
Insurance type	
Medicare advantage (%)	11,460 (80.6%)
Commercial (%)	2,755 (19.4%)
Patient outcomes	
Mean length of stay in days (Median, SD)	10.2 (6, 12.6)
Length of stay ≥1 day and Died (%)	1,716 (12.7%)
Length of stay ≥8 day and Died (%)	843 (5.9%)
Length of stay ≥15 day and Died (%)	436 (3.1%)
Length of stay ≥22 day and Died (%)	234 (1.6%)
Length of stay ≥29 day and Died (%)	124 (0.9%)

### Reporting summary

Further information on research design is available in the Nature Research Reporting Summary linked to this article.

## Supplementary information

Supplementary material

Reporting Summary

## Data Availability

Data for this study are not publicly available due to patient privacy concerns. The data that support the findings of this study are available from the corresponding author upon request.

## Code availability

The R and C codes are available at https://github.com/Penncil/dCLR and through our R package (version 1.0–2): ‘pda’ at https://cran.r-project.org/web/packages/pda/index.html.
