# Supplementary material for: Distributed learning for heterogeneous clinical data with application to integrating COVID-19 data across 230 sites
Source: NPJ Digit Med. 2022 Jun 14;5:76. doi: 10.1038/s41746-022-00615-8 (PMC9198031; doi:10.1038/s41746-022-00615-8)
Supplement: Supplementary file 1 — Supplementary material [file 41746_2022_615_MOESM1_ESM.docx]

**Supplementary Material to: Distributed learning for heterogeneous clinical data with application to integrating COVID-19 data across 230 sites**

Jiayi Tong^1^, Chongliang Luo^2^, Md Nazmul Islam^3^, Natalie E. Sheils^3^, John Buresh^3^, Mackenzie Edmondson^1^, Ebbing Lautenbach^1^, Peter A. Merkel^1^, Rui Duan^4^, Yong Chen^1*^

April 26, 2022

Summary

In this supplemental file, we provide the following materials:

Supplementary Method:

1): The proposed distributed conditional logistic regression (dCLR) algorithm

2): Simulation settings

3): Statistical Inference and Proof

Supplementary Notes:

4): Database Quality

Supplementary Figure:

5): Diagram of patient inclusion-exclusion criteria

## Supplementary Method 1. The proposed distributed conditional logistic regression (dCLR) algorithm

Suppose we have K different clinical sites. To keep the notation simple, we assume that each site has an equal number of n patients. Let {$x_{ij}$} denote the collection of risk factors and {$y_{ij}$} denote the independent response variable for the j-th patient in the i-th site where $j=1,...,n$ and $i=1,...,K$. The logistic regression model to characterize the association between the risk factors and the outcome is

$logit\left\{ Pr(y_{ij}=1|x_{ij}) \right\}=\alpha_{i}+\beta x_{ij}$ (1)

where $logit\left( p \right)=log\{p/(1-p)\}$, $\alpha_{i}$ represents the site-specific prevalence of response variable, and $\beta$ is the log odds ratio, meaning the association between risk factors $x_{ij}$ and the outcome $y_{ij}$.

Following Breslow and Day (1980)’s conditional logistic regression and Liang (1987)’s extended Mantel-Haenszel regression, the pairwise likelihood can be constructed by conditioning $(y_{ij,}y_{il})$ on their order statistics. The pairwise likelihood for the i-th site can be written as

$L_{i}\left( \beta\right)= \prod_{1\leq j<l\leq n} \left[ 1+exp\{-\left( y_{ij}-y_{il} \right)\left( x_{ij}-x_{il} \right)^{T}\beta\} \right]^{-1}$ (2)

We note that unlike generalized linear mixed effect model, where site-specific effects $\alpha_{i}$’s are assumed to follow a known distribution, the conditional pairwise likelihood eliminates the nuisance parameters $(\alpha_{1},...,\alpha_{K})$ through the conditioning technique, hence avoids estimation of the nuisance parameters. Moreover, as studied in Liang (1987), the estimator, defined as the maximum of the pairwise likelihood, retains high statistical efficiency.

Now summing over all K sites, the overall likelihood function can be written as the product of $L_{i}$ ,

$L^{*}\left( \beta\right)=\prod_{i=1}^{K} L_{i}\left( \beta\right)=\prod_{i=1}^{K} \prod_{1\leq j<l\leq n} \left[ 1+exp\{-\left( y_{ij}-y_{il} \right)\left( x_{ij}-x_{il} \right)^{T}\beta\} \right]^{-1}$ (3)

which can be calculated if we have access to the patient-level data from all sites.

However, in practice, the individual patient-level data are only available at the local site and for the rest of the clinical sites in the network, we can only access aggregated information. Motivated by the surrogate likelihood in Jordan et al. (2019) which approximates the target pairwise likelihood by the likelihood from a single site, we propose a surrogate pairwise likelihood which can still handle the heterogeneity across the clinical sites.

For simplicity, we assume the first site as the local site, where we have access to the individual patient-level data. Let $l_{1}(\beta)$ denote the pairwise log-likelihood function for the local site, $l_{1}\left( \beta\right)=\sum_{j<l} [1+exp\{-\left( y_{1j}-y_{1l} \right)\left( x_{1j}-x_{1l} \right)^{T}\beta\}]^{-1}/\binom{n}{2}$. We construct the following surrogate log pairwise likelihood function $\tilde{l}_{1}(\beta)$ with the patient-level data from the local site, the initial value $\bar{\beta}$, and the aggregated information $\nabla l_{i}(\bar{\beta})$ and $\nabla^{2}l_{i}(\bar{\beta})$. Specifically, we define

$\tilde{l_{1}}(\beta)=l_{1}(\beta)+\{\nabla l(\bar{\beta})-\nabla l_{1}(\bar{\beta})\}(\beta-\bar{\beta})+\frac{(\beta-\bar{\beta})^{T}\{\nabla^{2}l(\bar{\beta})-\nabla^{2}l_{1}(\bar{\beta})\}(\beta-\bar{\beta})}{2}$ (4)

where $l_{1}\left( \beta\right)$ is the log pairwise likelihood function calculated from patient-level data in the local site; $\nabla^{m}l(\bar{\beta})=K^{-1}\sum_{i=1}^{K} \nabla^{m}l_{i}(\bar{\beta}), m = 1, 2$ and ${\{\nabla l_{i}(\bar{\beta})\}}_{i=1,\ldots K}$, $\{{\nabla^{2}l_{i}(\bar{\beta})\}}_{i=1,\ldots,K}$ are the first and second gradients of the surrogate pairwise likelihood function at $\bar{\beta}$ respectively. By maximizing the surrogate pairwise likelihood $\tilde{l}_{1}(\beta)$ we obtain the surrogate estimator $\tilde{\beta}$.

A natural choice of the initial value of $\bar{\beta}$ is the maximum likelihood estimator of the local site $l_{1}\left( \beta\right)$. Alternatively, since the performance of the surrogate estimator $\tilde{\beta}$ may depend on the choice of the initial values, we may use the inverse variance weighted average of the estimates from all sites, i.e.,

$\bar{\beta}=(\sum_{i=1}^{K} {\hat{V}_{i}}^{-1})^{-1}\sum_{i=1}^{K} {\hat{V}_{i}}^{-1}\bar{\beta}_{i}$ (5)

where $\bar{\beta}_{i}$ is the maximum of the pairwise likelihood and $\hat{V}_{i}={\hat{\Sigma}_{1,i}}^{-1}(\bar{\beta}_{i})\hat{\Sigma}_{2,i}(\bar{\beta}_{i}){\hat{\Sigma}_{1,i}}^{-1}(\bar{\beta}_{i})/\binom{n}{2}$ is the covariance matrix of $\bar{\beta}_{i}$ in the i-th site; $\hat{\Sigma}_{1,i}(\beta)$ and $\hat{\Sigma}_{2,i}(\beta)$ are functions of $\beta$ for the i-th site. The definition of the covariance matrix, the asymptotic distribution of the surrogate estimator $\tilde{\beta}$, the derivation of the limiting distribution of $\tilde{\beta}$ are provided in Supplementary Appendix 1.

**Pseudo-code of the distributed conditional logistic regression (dCLR) algorithm**

**The dCLR algorithm:**

**Input:** Patient-level data $\{x_{ij}\}$ and $\{y_{ij}\}$, where $i$ denotes site index and $j$ the observation index, where $i=1,\ldots,K$ and $j=1,\ldots,n$. Note that $\{x_{ij}\}$ and $\{y_{ij}\}$ are stored in the i-th site locally.

**Output:** Estimator $\tilde{\beta}$ of the association between $\{x_{ij}\}$ and $\{y_{ij}\}$.

1: Obtain $\bar{\beta}_{i}=argmax l_{i}(\beta)$ and $\hat{V}_{i}$ with patient-level data in the i-th site

2: Broadcast $\bar{\beta}_{i}$ and $\hat{V}_{i}$ , and calculate initial value$\bar{\beta}$ with equation (5)

3: Suppose the 1^st^ site is the local site that we have access to the individual patient-level data

4: Transfer $\bar{\beta}$ to the local site

5: **for** i in c(1:K) do

6: Calculate$\nabla l_{i}(\bar{\beta})$, $\nabla^{2}l_{i}(\bar{\beta})$

7: Transfer the intermediate results to the local site

8: **end for**

9: Construct $\tilde{l}_{1}\left( \beta\right)$ as in equation (4) in the local site with $\bar{\beta}$,$\nabla l_{i}(\bar{\beta})$, $\nabla^{2}l_{i}(\bar{\beta})$

10: Obtain $\tilde{\beta}$ by maximizing $\tilde{l}_{1}\left( \beta\right)$

11: Calculate variance of $\tilde{\beta}$ with equation (A.4) and (A.5) in Supplementary Appendix 1

## Supplementary Method 2. Simulation settings

In our simulation study, we consider a setting where a binary outcome is associated with two risk factors, ($x_{1}, x_{2}$)*,* where $x_{1}$ represents a continuous predictor (e.g., age) and $x_{2}$ is a binary predictor (e.g., sex, race). The binary outcome Y (e.g., presence/absence of hospitalization) is generated from a Bernoulli distribution, with the conditional probability specified by the following logistic regression model,

$${logit\left\{ Pr(Y=1|x) \right\}= \beta_{0}+ \beta_{1}x_{1}+ \beta_{2}x}_{2}$$

where $\beta_{1}$ and $\beta_{2}$ are the coefficients of $x_{1}$ and $x_{2}$ respectively, and $\beta_{0}$ is the intercept, characterizing the prevalence of the outcome Y. We set the true value of $\beta_{1}$ is 1 and of $\beta_{2}$ is -1. The distribution of $x_{1}$ for each study site is $Uni(-1, 1)$ to mimic the empirical distribution of variable “age”, and $x_{2}$ is generated from a Bernoulli distribution with probability equal to 0.5 to mimic the empirical distribution of variable “sex”.

## Supplementary Method 3. Statistical Inference and Proof

***3.1 Theorem***

Let $\hat{\beta}$ denote the maximum pairwise likelihood function estimator of $L^{*}\left( \beta\right)$ defined in equation (3). The following proposition gives the asymptotic distribution of the pairwise likelihood estimator $\hat{\beta}$, which has been established in Liang (1987).

PROPOSITION 1: With $K$ fixed and $n$ increased, we have

$\sqrt{Kn}(\hat{\beta}-\beta)\to N(0, V)$ (A.1)

where $V={\Sigma_{1}}^{-1}\Sigma_{2}{\Sigma_{1}}^{-1}$, and

$\Sigma_{1}=E(\frac{\partial S^{(jl)}(\beta)}{\partial\beta}), \Sigma_{2}=cov\{S^{(jl)}(\beta)\}$ (A.2)

where $S^{(jl)}(\beta)=\sum_{i=1}^{K} {S_{i}}^{(jl)}(\beta)/K$and ${S_{i}}^{(jl)}(\beta)=(y_{ij}-y_{il})(x_{ij}-x_{il})exp\{-(y_{ij}-y_{il})(x_{ij}-x_{il})^{T}\beta\}/(1+exp\{-(y_{ij}-y_{il})(x_{ij}-x_{il})^{T}\beta\})$. Build on Liang (1987), we now provide the large sample distribution of the proposed surrogate estimator.

THEOREM 1: *As* $n$ *increases, given the initial estimator* $\bar{\beta}$ *satisfies* $||\bar{\beta}-\hat{\beta}||=O(n^{-1/2})$*, the proposed surrogate pairwise likelihood estimator* $\tilde{\beta}$ *satisfies*

$\sqrt{n}||\tilde{\beta}-\hat{\beta}||=O(n^{-1/2}) ||\bar{\beta}-\hat{\beta}||,$ (A.3)

*where* $\hat{\beta}$ *is the maximum pairwise likelihood estimator of* $L^{*}(\beta)$*.*

Since the pairs $\{\left( y_{ij,}y_{il} \right), 1\leq j<l\leq n\}$ consisting the pairwise likelihood are not i.i.d, the proof of Theorem 1 is based on the U statistics and is different from that of Jordan et al. (2019)

REMARK 1: Theorem 1 implies that the surrogate estimate $\tilde{\beta}$ has the same limiting distribution as $\hat{\beta}$. In a distributed setting, the variance of $\tilde{\beta}$ can be consistently estimated by its empirical estimator ${\hat{\Sigma}_{1,i=1}}^{-1}(\tilde{\beta})\hat{\Sigma}_{1,i=1}(\tilde{\beta}){\hat{\Sigma}_{1,i=1}}^{-1}(\tilde{\beta})/\binom{n}{2}$ with the patient-level data from the local site (i.e., site 1; i = 1), where and are function of $\beta$for the i-th site,

$\hat{\Sigma}_{1,i}\left( \beta\right)={\binom{n}{2}}^{-1}\sum_{j<l} -\frac{\partial{S_{i}}^{(jl)}(\beta)}{\partial\beta}$, (A.4)

$\hat{\Sigma}_{2,i}(\beta)=\frac{4}{n(n-1)(n-2)}\sum_{j<l} {S_{i}}^{(jl)}(\beta){S_{i}}^{(jl)}(\beta)^{T}$, (A.5)

There is no extra communication across the sites after we obtain the $\tilde{\beta}$ in the calculation of variance of $\tilde{\beta}$.

- 1. ***Proof of Theorem 1***

In the proof below, we rewrite the log-conditional likelihood term

$$\mathcal{l}_{i}^{jl}=\log\left\{ 1+\exp\left( \left( y_{ij}-y_{il} \right)\left( x_{ij}-x_{il} \right)\beta^{T} \right) \right\}.$$

We denote $\|.\|$ the L-2 norm for a vector or Frobenius norm for a matrix. Before proving Theorem 1, we first introduce the regularity conditions and some corollaries. First of all, we present a result about the Lipschitz continuity of $\nabla^{2}\mathcal{l}_{i}^{jl}$.

COROLLARY 1: *For any* $i, j, \beta,\beta'$*, we have*

$$\|\nabla^{2}\mathcal{l}_{i}^{jl}\left( \beta^{'} \right)-\nabla^{2}\mathcal{l}_{i}^{jl}\left( \beta\right)\|\leq L(X_{ij}, Y_{ij}, X_{il}, Y_{il})\|\beta^{'}-\beta\|$$

*where* $L(X_{ij}, Y_{ij}, X_{il}, Y_{il})$ *is given by*

$$L\left( X_{ij}, Y_{ij}, X_{il}, Y_{il} \right)=\left\| Y_{ij}-Y_{il} \right\|^{3}\left\| X_{ij}-X_{il} \right\|^{3}.$$

*Proof:* Based on the likelihood, we have

$$\nabla^{2}\mathcal{l}_{i}^{jl}\left( \beta^{'} \right)-\nabla^{2}\mathcal{l}_{i}^{jl}\left( \beta\right)=\left\{ \frac{T^{'}}{\left( 1+T^{'} \right)^{2}}-\frac{T}{\left( 1+T \right)^{2}} \right\}\left( y_{ij}-y_{il} \right)^{2}\left( x_{ij}-x_{il} \right)^{\bigotimes2}$$

where $T^{'}=exp(\left( y_{ij}-y_{il} \right)\left( x_{ij}-x_{il} \right)^{'}\beta')$ and $T=exp(\left( y_{ij}-y_{il} \right)\left( x_{ij}-x_{il} \right)^{'}\beta)$. So,

$$\left\| \nabla^{2}\mathcal{l}_{i}^{jl}\left( \beta^{'} \right)-\nabla^{2}\mathcal{l}_{i}^{jl}\left( \beta\right) \right\|$$

$$\leq\left| \frac{T^{'}}{\left( 1+T^{'} \right)^{2}}-\frac{T}{\left( 1+T \right)^{2}} \right|\left\| \left( y_{ij}-y_{il} \right)^{2}\left( x_{ij}-x_{il} \right)^{\bigotimes2} \right\|$$

$$=\left\| \left( y_{ij}-y_{il} \right)^{2}\left( x_{ij}-x_{il} \right)^{\bigotimes2} \right\|\left| \frac{{(T}^{'}-T)(1-T'T)}{\left( 1+T^{'} \right)^{2}\left( 1+T \right)^{2}} \right|$$

$$\leq\left\| \left( y_{ij}-y_{il} \right)^{2}\left( x_{ij}-x_{il} \right)^{\bigotimes2} \right\|\left| \frac{{(T}^{'}-T)}{\left( 1+T^{'} \right)\left( 1+T \right)} \right|\left| \frac{\left( 1-T^{'}T \right)}{\left( 1+T^{'} \right)\left( 1+T \right)} \right|$$

$$\leq\left\| \left( y_{ij}-y_{il} \right)^{2}\left( x_{ij}-x_{il} \right)^{\bigotimes2} \right\|\left| \frac{{(T}^{'}-T)}{\left( 1+T^{'} \right)\left( 1+T \right)} \right|$$

$$\leq\left| y_{ij}-y_{il} \right|^{3}\|{x_{ij}-x_{il}\left. \right\|}^{3}\left| \frac{T''}{\left( 1+T^{'} \right)\left( 1+T \right)} \right|\|\beta^{'}-\bar{\beta}\|$$

with

$$T^{''}=\exp\left( \left( y_{ij}-y_{il} \right)\left( x_{ij}-x_{il} \right)^{'}\beta^{''} \right)=\left( T^{'} \right)^{a^{'}}\left( T \right)^{1-a'}\leq\max\left\{ T^{'}, T \right\},$$

and $\beta^{''}=a^{'}\beta^{'}+\left( 1-a^{'} \right)\beta$ is the linear combination of $\beta$ and $\beta'$. $\beta''$ is between $\beta$ and $\beta'$, $0\leq a'\leq1$. Thus,

##

$$\left\| \boldsymbol{\nabla}^{\boldsymbol{2}}\mathcal{l}_{\boldsymbol{i}}^{\boldsymbol{jl}}\left( \boldsymbol{\beta}^{\boldsymbol{'}} \right)\boldsymbol{-}\boldsymbol{\nabla}^{\boldsymbol{2}}\mathcal{l}_{\boldsymbol{i}}^{\boldsymbol{jl}}\left( \boldsymbol{\beta} \right) \right\|\boldsymbol{\leq}\left| \boldsymbol{y}_{\boldsymbol{ij}}\boldsymbol{-}\boldsymbol{y}_{\boldsymbol{il}} \right|^{\boldsymbol{3}}\boldsymbol{\|}{\boldsymbol{x}_{\boldsymbol{ij}}\boldsymbol{-}\boldsymbol{x}_{\boldsymbol{il}}\left. \boldsymbol{} \right\|}^{\mathbf{3}}\boldsymbol{\|}\boldsymbol{\beta}^{\boldsymbol{'}}\boldsymbol{-}\bar{\boldsymbol{\beta}}\boldsymbol{\|}$$

Then, we introduce the following conditions for Theorem 1.

Condition 1 (**Local convexity**): The Hessian matrix $I_{i}\left( \beta\right)=\mathbb{E}_{i}[\nabla^{2}\mathcal{l}_{j}(\beta)]$ of the pairwise log-likelihood function $\mathcal{l}_{i}$ at site I is invertible at  $\hat{\beta}$, there exist positive constant $\lambda$, such as $\lambda I_{p}≼\nabla^{2}\mathcal{l}_{j}(\beta)$.

Condition 2 (**Smoothness**): There exist constants $(G, H)$ such that $\mathbb{E}\left\| \nabla\mathcal{l}_{j}^{1,2}\left( \beta\right) \right\|_{2}^{4}\leq G^{4}$, $\mathbb{E}\left\| \nabla\mathcal{l}_{j}^{1,2}\left( \beta\right)-I_{i}(\beta) \right\|_{2}^{4}\leq H^{4}$ for all $k$ and $\beta\in U\left( \rho\right)=\{\beta:\|\beta-\hat{\beta}\}\leq\rho$. For the function $L\left( X_{1}, Y_{1}, X_{2}, Y_{2} \right)$ in Corollary 1, there exist constant L such that $\mathbb{E}_{i}[L\left( X_{1}, Y_{1}, X_{2}, Y_{2} \right)^{2}]<L^{2}$ and $\mathbb{E}_{i}[\left\{ L\left( X_{1}, Y_{1}, X_{2}, Y_{2} \right)-\mathbb{E}_{i}\left[ L\left( X_{1}, Y_{1}, X_{2}, Y_{2} \right) \right] \right\}^{2}]<L^{2}$.

COROLLARY 2: *The following inequality holds with probability* $1-O(n^{-1})$*.*

$$\frac{1}{K\left( \begin{matrix} n \\ 2 \end{matrix} \right)}\sum_{i, j, l} L\left( X_{ij}, Y_{ij}, X_{il}, Y_{il} \right)\leq4\mathbb{E}L\left( X_{ij}, Y_{ij}, X_{il}, Y_{il} \right)+6L$$

*Proof:* Define  $\tilde{L}\left( X_{ij}, Y_{ij}, X_{il}, Y_{il} \right)=L\left( X_{ij}, Y_{ij}, X_{il}, Y_{il} \right)\mathbb{-E}L\left( X_{ij}, Y_{ij}, X_{il}, Y_{il} \right)$. Now we calculate the following probability of the inequality using Markov inequality:

$$\mathbb{P}\left( \frac{1}{K\left( \begin{matrix} n \\ 2 \end{matrix} \right)}\sum_{i, j, l} L\left( X_{ij}, Y_{ij}, X_{il}, Y_{il} \right)>4\mathbb{E}L\left( X_{ij}, Y_{ij}, X_{il}, Y_{il} \right)+6L \right)$$

$$\mathbb{=P}\left( \frac{1}{K\left( \begin{matrix} n \\ 2 \end{matrix} \right)}\sum_{i, j, l} \tilde{L}\left( X_{ij}, Y_{ij}, X_{il}, Y_{il} \right)>3\mathbb{E}L\left( X_{ij}, Y_{ij}, X_{il}, Y_{il} \right)+6L \right)$$

$$\leq\left\{ 3\mathbb{E}L\left( X_{ij}, Y_{ij}, X_{il}, Y_{il} \right)+6L \right\}^{-2}Var\left( \frac{1}{K\left( \begin{matrix} n \\ 2 \end{matrix} \right)}\sum_{i, j, l} \tilde{L}\left( X_{ij}, Y_{ij}, X_{il}, Y_{il} \right) \right)$$

$$\leq\left\{ 3\mathbb{E}L\left( X_{ij}, Y_{ij}, X_{il}, Y_{il} \right)+6L \right\}^{-2}\frac{4}{K^{2}n^{2}\left( n-1 \right)^{2}}\{\sum_{i, j, l} Var\left( \tilde{L}\left( X_{ij}, Y_{ij}, X_{il}, Y_{il} \right) \right)+\sum_{i, j, l,l'} Cov\left( \tilde{L}\left( X_{ij}, Y_{ij}, X_{il}, Y_{il} \right),\tilde{L}\left( X_{ij}, Y_{ij}, X_{il^{'}}, Y_{il^{'}} \right) \right)\}$$

Now, let’s prove Theorem 1.

*Proof*. Define the “good event”

$$\mathcal{E}_{0}=\{\frac{1}{\left( \begin{matrix} n \\ 2 \end{matrix} \right)}\sum L\left( X_{i}, X_{j}, Y_{i}, Y_{j} \right)\leq2L\},$$

$$\mathcal{E}_{1}=\{\|\nabla^{2} {\tilde{\mathcal{l}}}_{1}\left( \hat{\beta} \right)-\nabla^{2}\mathcal{l}\left( \beta\right)\|\leq\frac{\rho\lambda}{2}\},$$

$$\mathcal{E}_{2}=\{\|\nabla{\tilde{\mathcal{l}}}_{1}\left( \hat{\beta} \right)\|\leq\frac{\left( 1-\rho\right)\lambda\delta_{p}}{4}\}$$

By Lemma 6 in Zhang et al. (2013), we have under event $\mathcal{E=}\mathcal{E}_{0}\cap\mathcal{E}_{1}\cap\mathcal{E}_{2}$, $\|\bar{\beta}-\hat{\beta}\|\leq\frac{2\|\nabla{\tilde{\mathcal{l}}}_{1}\left( \hat{\beta} \right)\|}{\left( 1-\rho\right)\lambda}$ .

We then prove that $\left\| \nabla{\tilde{\mathcal{l}}}_{1}\left( \hat{\beta} \right) \right\|=O\left( n^{-\frac{1}{2}} \right)\|\bar{\beta}-\hat{\beta}\|$.

$$\nabla{\tilde{\mathcal{l}}}_{1}\left( \hat{\beta} \right)= \nabla\mathcal{l}_{1}\left( \hat{\beta} \right)+\left\{ \nabla\mathcal{l}\left( \bar{\beta} \right)-\nabla\mathcal{l}_{1}\left( \bar{\beta} \right) \right\}+\left\{ \nabla^{2}\mathcal{l}\left( \bar{\beta} \right)-\nabla^{2}\mathcal{l}_{1}\left( \bar{\beta} \right) \right\}\left( \hat{\beta}-\bar{\beta} \right)-\nabla\mathcal{l}\left( \hat{\beta} \right)$$

$=\left\{ \nabla\mathcal{l}_{1}\left( \hat{\beta} \right)-\nabla\mathcal{l}_{1}\left( \bar{\beta} \right) \right\}+\left\{ \nabla\mathcal{l}\left( \hat{\beta} \right)-\nabla\mathcal{l}\left( \bar{\beta} \right) \right\}+\left\{ \nabla^{2}\mathcal{l}\left( \bar{\beta} \right)-\nabla^{2}\mathcal{l}_{1}\left( \bar{\beta} \right) \right\}\left( \hat{\beta}-\bar{\beta} \right)$

$$=-\nabla^{2}\mathcal{l}_{1}\left( \beta^{'} \right)\left( \bar{\beta}-\hat{\beta} \right)+\nabla^{2}\mathcal{l}\left( \beta^{'} \right)\left( \bar{\beta}-\hat{\beta} \right)-\{\nabla^{2}\mathcal{l}\left( \bar{\beta} \right)-\nabla^{2}\mathcal{l}_{1}(\bar{\beta})\}(\bar{\beta}-\hat{\beta})$$

$$=\{\nabla^{2}\mathcal{l}\left( \beta^{'} \right)-\nabla^{2}\mathcal{l}_{1}\left( \beta^{'} \right)-\nabla^{2}\mathcal{l}\left( \bar{\beta} \right)+\nabla^{2}\mathcal{l}_{1}(\bar{\beta})\}(\bar{\beta}-\hat{\beta})$$

where $\beta^{'}=a \hat{\beta}+\left( 1-a \right) \bar{\beta}, 0\leq a\leq1.$ The first equation is due to the definition of  ${\tilde{\mathcal{l}}}_{1}$ and $\nabla\mathcal{l}\left( \hat{\beta} \right)=0$. Therefore,

$$\left\| \nabla{\tilde{\mathcal{l}}}_{1}\left( \hat{\beta} \right) \right\|\leq\{\left\| \nabla^{2}\mathcal{l}\left( \beta^{'} \right)-\nabla^{2}\mathcal{l}\left( \bar{\beta} \right) \right\|+\|\nabla^{2}\mathcal{l}_{1}\left( \beta^{'} \right)-\nabla^{2}\mathcal{l}_{1}\left( \bar{\beta} \right)\|\}\|\bar{\beta}-\hat{\beta}\|$$

Based on the smoothness Condition 2 and Corollary 2, we have

$$\left\| \nabla{\tilde{\mathcal{l}}}_{1}\left( \hat{\beta} \right) \right\|\leq\left\{ \left\| \sum_{i,j,l} \frac{1}{K\left( \begin{matrix} n \\ 2 \end{matrix} \right)} \left[ \nabla^{2}\mathcal{l}_{i}^{jl}\left( \beta^{'} \right)-\nabla^{2}\mathcal{l}_{i}^{jl}\left( \bar{\beta} \right) \right] \right\|+\left\| \sum_{j,l} \frac{1}{\left( \begin{matrix} n \\ 2 \end{matrix} \right)} \left[ \nabla^{2}\mathcal{l}_{1}^{jl}\left( \beta^{'} \right)-\nabla^{2}\mathcal{l}_{1}^{jl}\left( \bar{\beta} \right) \right] \right\| \right\}\left\| \bar{\beta}-\hat{\beta} \right\|$$

$$\leq[\frac{1}{K\left( \begin{matrix} n \\ 2 \end{matrix} \right)}\sum_{i,j,l} L\left( X_{ij}, Y_{ij}, X_{il}, Y_{il} \right)+\frac{1}{\left( \begin{matrix} n \\ 2 \end{matrix} \right)}\sum_{j,l} L\left( X_{ij}, Y_{ij}, X_{il}, Y_{il} \right)]\left\| \beta^{'}-\bar{\beta} \right\|\|\bar{\beta}-\hat{\beta}\|$$

$$\leq\left( 4\mathbb{E}L\left( X_{ij}, Y_{ij}, X_{il}, Y_{il} \right)+6L \right)\left\| \beta^{'}-\bar{\beta} \right\|\left\| \bar{\beta}-\hat{\beta} \right\|$$

$$=O(n^{-1/2})\left\| \bar{\beta}-\hat{\beta} \right\|$$

We get the last equation because $\left\| \beta^{'}-\bar{\beta} \right\|\leq\left\| \bar{\beta}-\hat{\beta} \right\|=O(n^{-1/2})$. Then, we need to prove the high probability bound of the “good events”. First, we investigate the event $\mathcal{E}_{0}$. The probability of the $\mathcal{E}_{0}^{c}$ can be calculated with Markov inequality as follows:

$$\mathbb{P}\left( \mathcal{E}_{0}^{c} \right)\mathbb{\leq P}\left( \frac{1}{\left( \begin{matrix} n \\ 2 \end{matrix} \right)}\sum_{j,l} \tilde{L}\left( X_{ij}, Y_{ij}, X_{il}, Y_{il} \right)\mathbb{>-E}L\left( X_{ij}, Y_{ij}, X_{il}, Y_{il} \right)+2L \right)$$

$$\leq\left[ \mathbb{-E}L\left( X_{ij}, Y_{ij}, X_{il}, Y_{il} \right)+2L \right]^{-2}Var\left( \frac{1}{\left( \begin{matrix} n \\ 2 \end{matrix} \right)}\sum_{j,l} \tilde{L}\left( X_{ij}, Y_{ij}, X_{il}, Y_{il} \right) \right)$$

$$\leq\left[ \mathbb{-E}L\left( X_{ij}, Y_{ij}, X_{il}, Y_{il} \right)+2L \right]^{-2}\frac{1}{n^{2}\left( n-1 \right)^{2}}\left\{ \sum_{j,l} Var\tilde{L}\left( X_{ij}, Y_{ij}, X_{il}, Y_{il} \right)+\sum_{j,l,l^{'}} Cov\left( \tilde{L}\left( X_{ij}, Y_{ij}, X_{il}, Y_{il} \right),\tilde{L}\left( X_{ij}, Y_{ij}, X_{il^{'}}, Y_{il^{'}} \right) \right) \right\}$$

$$\leq O(n^{-1})$$

We then investigate $\mathcal{E}_{1}$. Then, we have

$$\mathbb{P}\left( \mathcal{E}_{1}^{c} \right)\mathbb{=P}\left( \left\| \nabla^{2}\mathcal{l}_{1}\left( \hat{\beta} \right)+\nabla^{2}\mathcal{l}\left( \bar{\beta} \right)-\nabla^{2}\mathcal{l}_{1}\left( \bar{\beta} \right)-\nabla^{2}\mathcal{l}\left( \hat{\beta} \right) \right\|>\frac{\rho\lambda}{2} \right)$$

$$\mathbb{\leq P(}\frac{1}{\left( \begin{matrix} n \\ 2 \end{matrix} \right)}\|\sum_{j,l} \nabla^{2}\mathcal{l}_{1}^{jl}\left( \hat{\beta} \right)-\nabla^{2}\mathcal{l}_{1}^{jl}(\bar{\beta})\|>\frac{\rho\lambda}{4}\mathbb{)+P(}\frac{1}{K\left( \begin{matrix} n \\ 2 \end{matrix} \right)}\|\sum_{i,j,l} \nabla^{2}\mathcal{l}_{k}^{jl}\left( \hat{\beta} \right)-\nabla^{2}\mathcal{l}_{k}^{jl}(\bar{\beta})\|>\frac{\rho\lambda}{4})$$

Let $\beta^{'}=\hat{\beta}$ and $\beta=\bar{\beta}$ in Corollary 1, then we have

$$\left\| \nabla^{2}\mathcal{l}_{i}^{jl}\left( \hat{\beta} \right)-\nabla^{2}\mathcal{l}_{i}^{jl}\left( \bar{\beta} \right) \right\|\leq L(X_{ij}, Y_{ij},X_{il}, Y_{il})\|\hat{\beta}-\bar{\beta}\|$$

In the above function, the first term has the following probability for large enough n,

$$\mathbb{P}\left( \frac{1}{\left( \begin{matrix} n \\ 2 \end{matrix} \right)}\left\| \sum_{j,l} \nabla^{2}\mathcal{l}_{1}^{jl}\left( \hat{\beta} \right)-\nabla^{2}\mathcal{l}_{1}^{jl}\left( \bar{\beta} \right) \right\|>\frac{\rho\lambda}{4} \right)$$

$$\mathbb{\leq P}\left( \frac{1}{\left( \begin{matrix} n \\ 2 \end{matrix} \right)}\sum_{j,l} \left\| \nabla^{2}\mathcal{l}_{1}^{jl}\left( \hat{\beta} \right)-\nabla^{2}\mathcal{l}_{1}^{jl}\left( \bar{\beta} \right) \right\|>\frac{\rho\lambda}{4} \right)$$

$$\mathbb{\leq P}\left( \frac{1}{\left( \begin{matrix} n \\ 2 \end{matrix} \right)}\sum_{j,l} L\left( X_{1j}, Y_{1j}, X_{1l}, Y_{1l} \right)>\frac{\rho\lambda}{4\left\| \hat{\beta}-\bar{\beta} \right\|} \right)$$

$$\mathbb{\leq P}\left( \frac{1}{\left( \begin{matrix} n \\ 2 \end{matrix} \right)}\sum_{j,l} L\left( X_{1j}, Y_{1j}, X_{1l}, Y_{1l} \right)>2L \right)$$

$$\mathbb{= P}\left( \mathcal{E}_{0}^{c} \right)=O(n^{-1})$$

Similarly, for the second term, we have:

$$\mathbb{P}\left( \frac{1}{K\left( \begin{matrix} n \\ 2 \end{matrix} \right)}\left\| \sum_{i,j,l} \nabla^{2}\mathcal{l}_{i}^{jl}\left( \hat{\beta} \right)-\nabla^{2}\mathcal{l}_{i}^{jl}\left( \bar{\beta} \right) \right\|>\frac{\rho\lambda}{4} \right)=O(n^{-1})$$

Thus, $\mathbb{P}\left( \mathcal{E}_{1}^{c} \right)=O(n^{-1})$. Finally, we can investigate $\mathcal{E}_{2}$.

$$\mathbb{P}\left( \mathcal{E}_{2}^{c} \right)\mathbb{=P}\left( \left\| \nabla{\tilde{\mathcal{l}}}_{1}\left( \hat{\beta} \right) \right\|>\frac{\left( 1-\rho\right)\lambda\delta_{p}}{4} \right)$$

$$\mathbb{\leq P}\left( \left\{ \left\| \nabla^{2}\mathcal{l}\left( \beta^{'} \right)-\nabla^{2}\mathcal{l}\left( \bar{\beta} \right) \right\|+\left\| \nabla^{2}\mathcal{l}_{1}\left( \beta^{'} \right)-\nabla^{2}\mathcal{l}_{1}\left( \bar{\beta} \right) \right\| \right\}\left\| \beta^{'}-\hat{\beta} \right\|>\frac{\left( 1-\rho\right)\lambda\delta_{p}}{4} \right)$$

$$\mathbb{\leq P}\left( \left\{ \left\| \nabla^{2}\mathcal{l}\left( \beta^{'} \right)-\nabla^{2}\mathcal{l}\left( \bar{\beta} \right) \right\| \right\}>\frac{\left( 1-\rho\right)\lambda\delta_{p}}{4\left\| \beta^{'}-\hat{\beta} \right\|} \right)\mathbb{+P}\left( \left\{ \left\| \nabla^{2}\mathcal{l}_{1}\left( \beta^{'} \right)-\nabla^{2}\mathcal{l}_{1}\left( \bar{\beta} \right) \right\| \right\}>\frac{\left( 1-\rho\right)\lambda\delta_{p}}{4\left\| \beta^{'}-\hat{\beta} \right\|} \right)$$

$\leq O(n^{-1}$)

## Supplementary Notes 4. Database Quality

# *4.1 Standardization of Data Entry and Data Structure*

Medical and pharmacy claims data are captured, predominantly electronically, from sites of care seeking third-party reimbursement for both Medicare and commercial plans using the industry standard data collection forms HCFA/CMS-1500 for facility claims, UB04/CMS-1450 for professional services and outpatient claims, and NCPDP for pharmacy claims or their electronic equivalents. Structured data from these standardized forms are coded using the International Classification of Diseases, Tenth Revision, Clinical Modification (ICD-10-CM), National Drug Codes (NDC), Current Procedural Terminology (CPT) codes, and Logical Observation Identifiers Names and Codes (LOINC) codes, and Diagnosis Related Groups (DRG). This nomenclature ensures consistency of data collection across geographic regions, health systems, and payers throughout the United States.

# *4.2 Methods to Control for Errors in Sampling and Data Collection*

Claims that do not adhere to the form or coding standards described above are rejected from reimbursement, minimizing the risk that inappropriately structured data are included in the database. Data specific to SARS-CoV-2 and COVID-19 has an additional Quality Control layer to control for errors in sampling and data collection; this is described below in the section on Quality Control.

# *4.3 Data Relevance and Accuracy*

Data are transferred into the UnitedHealth Group (UHG) Clinical Discovery Database, where a dedicated team pursues data management to ensure accurate matching of source data to an individual. This protocol uses unique identifiers to match them to existing identifiers in the UHG Clinical Discovery Database to determine whether the individual already exists in the platform. A unique identification number is generated for each individual so that data from multiple sources can be linked back to that identification number. Individuals that fail to meet the matching criteria are excluded from the UHG Clinical Discovery Database to reduce the risk of erroneous linkage of records. Those whose claims do not fulfill basic standardized data structure requirements described previously are also excluded. During this, all member protected data are stored in a separate database that is only accessible by a designated engineering team. In addition to a persistent identifier being generated for each member, a de-identified primary key is also generated. The de-identified primary key is recycled every 6 months, at which time each member is assigned a new de-identified primary key. Data that are made available for research through the UHG Clinical Discovery Database use the de-identified primary key as the link across data tables. All protected information has been removed, ensuring any research performed is limited to retrospective analysis of de-identified data and accessed in accordance with Health Insurance Portability and Accountability Act regulations.

# *4.4 Sufficiency of Basic Data*

As described above, individuals lacking enough data to be assigned a unique primary key are excluded from the UHG Clinical Discovery Database, as are patients whose claims did not fulfill basic data structure requirements. In a given month in 2019, the UHG Clinical Discovery Database contained one or more claims from 5 million Medicare Advantage enrollees and 20 million commercially insured individuals. Further information on data sufficiency for the research performed in this manuscript can be found in **Figure 3** in the manuscript.

# *4.5 Adequacy of Possible Derived Data*

To reduce the risk of introducing error to standardized, structured claims data, derivation of source data within the UHG Clinical Discovery Database is minimal. The Data Integration team loads, formats, and join the data to appropriate dimension tables. Dimension tables are combined with raw claims information to limit the number of times external tables need to be referenced. Researchers may request derived fields within data tables prepared specifically for a project. This process is managed by the Data Enrichment team, who creates data dictionaries to accompany derived fields. Tables containing derived data are stored separately from raw source data.

# *4.6 Design of Computer Editing Methods*

Access to modify/edit source data is restricted to a subset of data specialists. Each step in the data flow has a restricted list of individuals able to perform any type of editing to the database, and access level varies by team (Data Integration, Data Enrichment). Researchers using the Clinical Discovery Database may not edit any source data or enrichment data. They are instead given access to “sandbox” locations where they may request editing access for the data tables used in their analyses.

# *4.7 Quality Control*

In addition to the quality control mechanisms described during the matching procedures to reject non-linkable or inappropriately structured data, a COVID-19 data source-specific layer of quality control is also present, given the rapidly evolving situation. SARS-CoV-2 lab tests included in the UHG Clinical Discovery Database exclude custom local codes or codes that are not present in the LOINC organization’s guidance for mapping SARS-CoV-2 and COVID-19 related LOINC terms. Test information provided via the LOINC code compliments the test type (antibody, RT-PCR, etc.) as well as the result value (detected, not detected, not given/cancelled). Suspected COVID-19 inpatient cases are manually reviewed daily by health plan clinical staff via clinical notes to determine an individual’s COVID-19 status. Each case is then manually flagged as either negative, confirmed, presumed positive, or needs clinical review. If a case is confirmed, it is not reviewed again. If a case is listed as negative or unknown, it is periodically reviewed for changes in the record. All others are reviewed and updated daily.

# *4.8 Differences Across Groups*

While the data for Medicare Advantage and commercially insured enrollees is processed in a similar manner, these groups are substantially different. First, there are systematic differences in patient characteristics, most remarkably the older age and the higher prevalence of all comorbidities. These differences are tabulated in the UHG Clinical Discovery Database, there are restrictions from individual employers on these use of data for research. Therefore, commercial insurance claims that are available for analyses are a subset of the overall commercially insured population.

# *4.9 Data Sharing*

The data are proprietary and are not available for public use but can be made available to editors and their approved auditors under a data use agreement to confirm the findings of the current study.

## Supplementary Figure. Diagram of patient inclusion-exclusion criteria


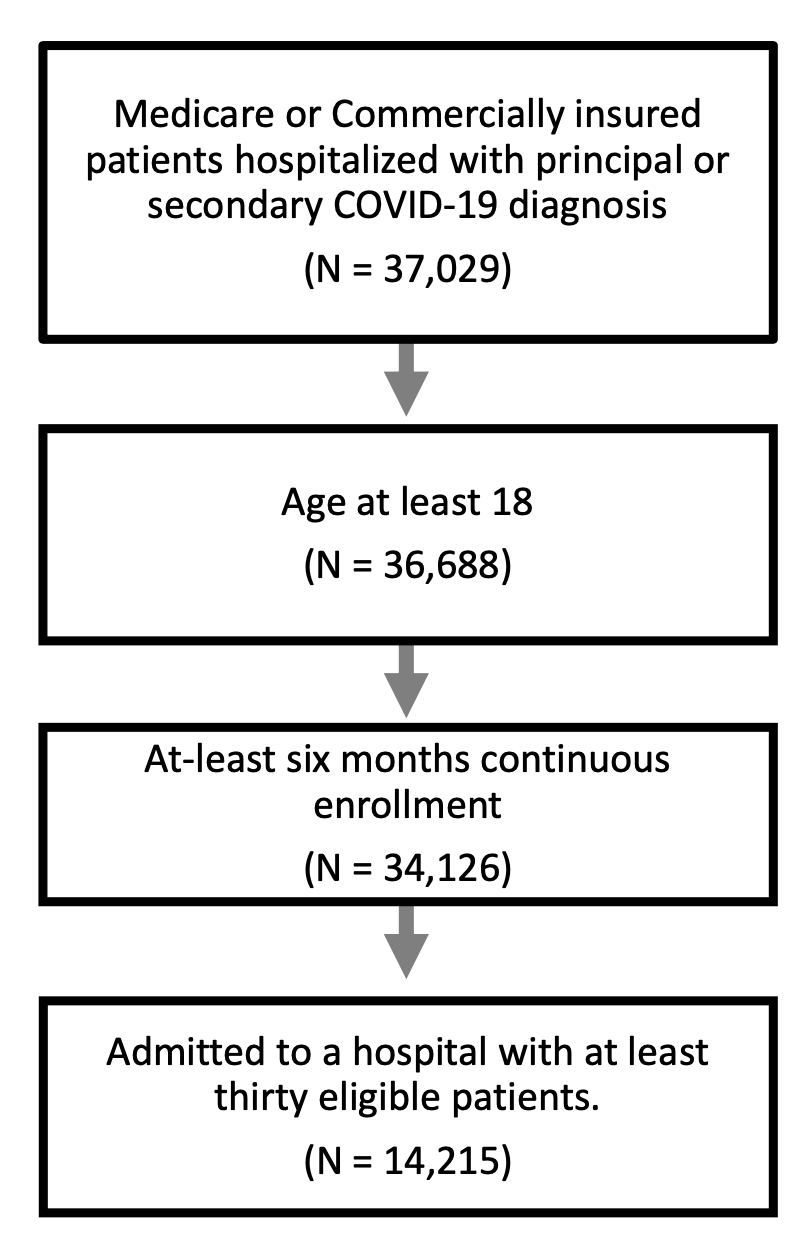


**Figure A1:** Diagram of the patient inclusion-exclusion criteria.
